# Supplementary material for: Synergistic Neuroprotection Through Epigenetic Modulation by Combined Curcumin-Enriched Turmeric Extract and L-Ascorbic Acid in Oxidative Stress-Induced SH-SY5Y Cell Damage
Source: Foods. 2025 Mar 5;14(5):892. doi: 10.3390/foods14050892 (PMC11898916; doi:10.3390/foods14050892)
Supplement: Supplementary file 1 [file foods-14-00892-s001.zip › foods-3507836-supplementary.pdf]

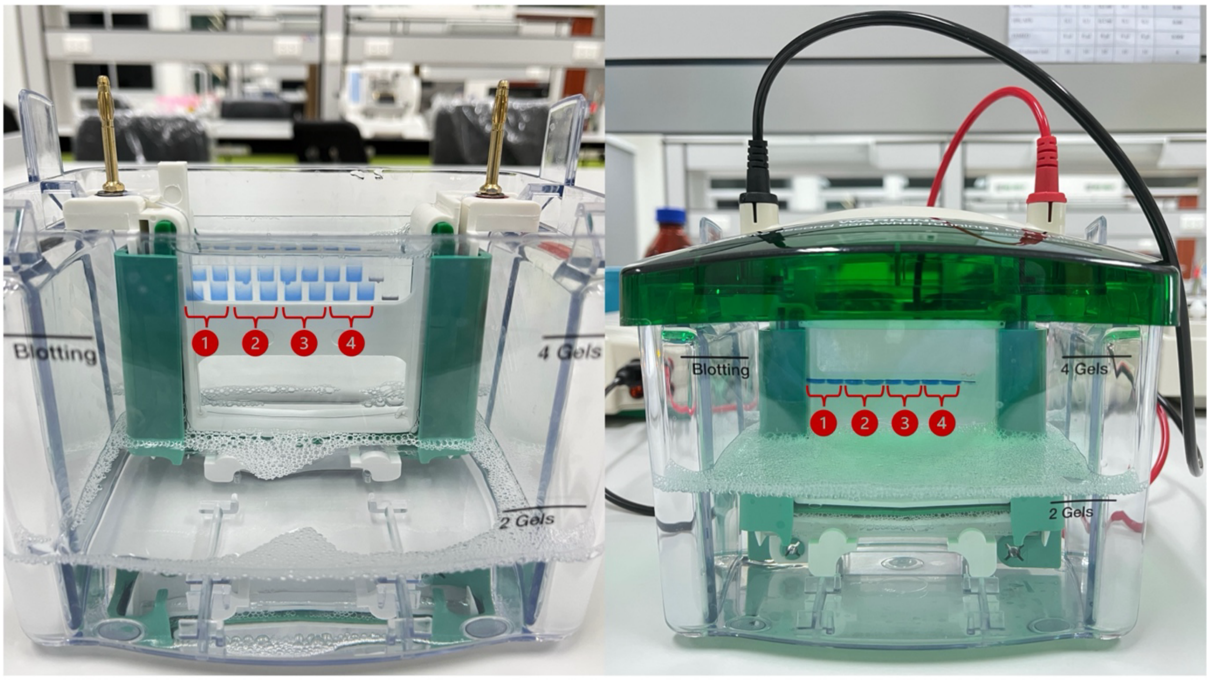

A B

Figure S1: Western blot analysis during (A) protein loading into the stacking gel and (B) separation through the resolving gel. Each experiment was performed in duplicate. The experimental groups included: (1) naïve control, (2) hydrogen peroxide + vehicle, (3) hydrogen peroxide + curcumin-enriched turmeric extract combined with L-ascorbic acid (20 μg/mL), and (4) hydrogen peroxide + curcumin-enriched turmeric extract combined with L-ascorbic acid (40 μg/mL).

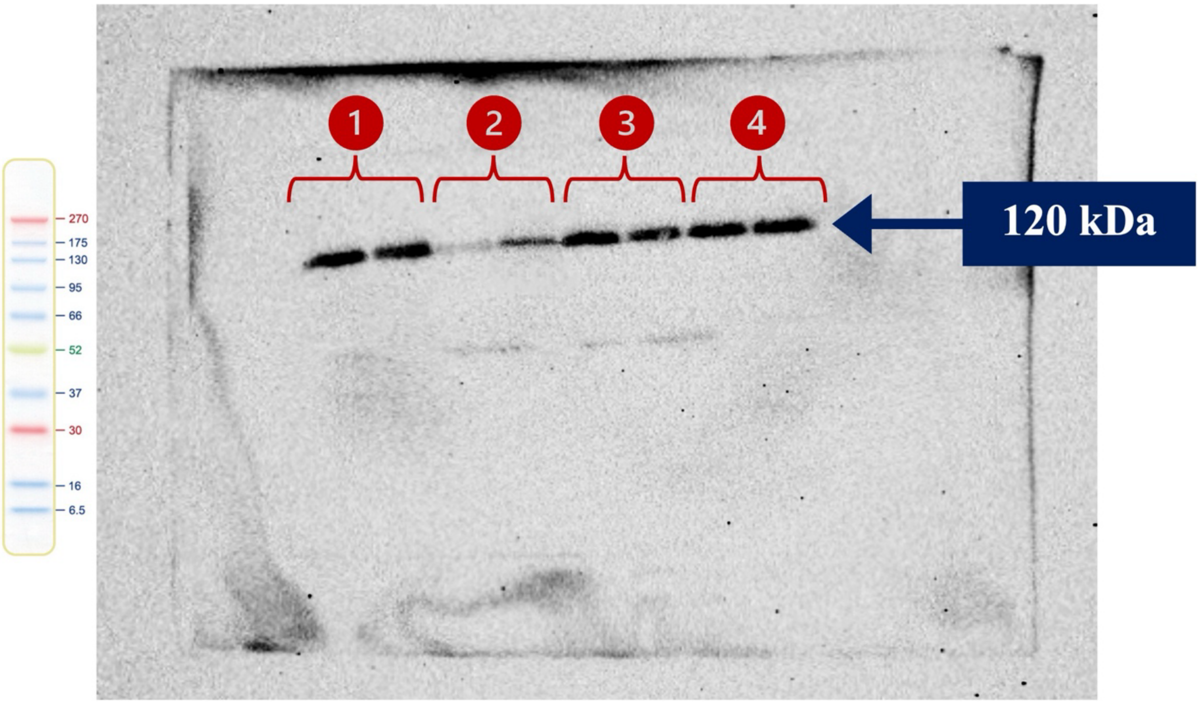

Figure S2: Western blot analysis of SIRT1 expression in SH-SY5Y cells following treatment with curcumin-enriched turmeric extract combined with L-ascorbic acid against hydrogen peroxide-induced neurotoxicity. The experimental groups included: (1) naïve control, (2) hydrogen peroxide + vehicle, (3) hydrogen peroxide + curcumin-enriched turmeric extract combined with L-ascorbic acid (20 μg/mL), and (4) hydrogen peroxide + curcumin-enriched turmeric extract combined with L-ascorbic acid (40 μg/mL). This figure presents the original, unprocessed, uncropped, full-length membrane images.

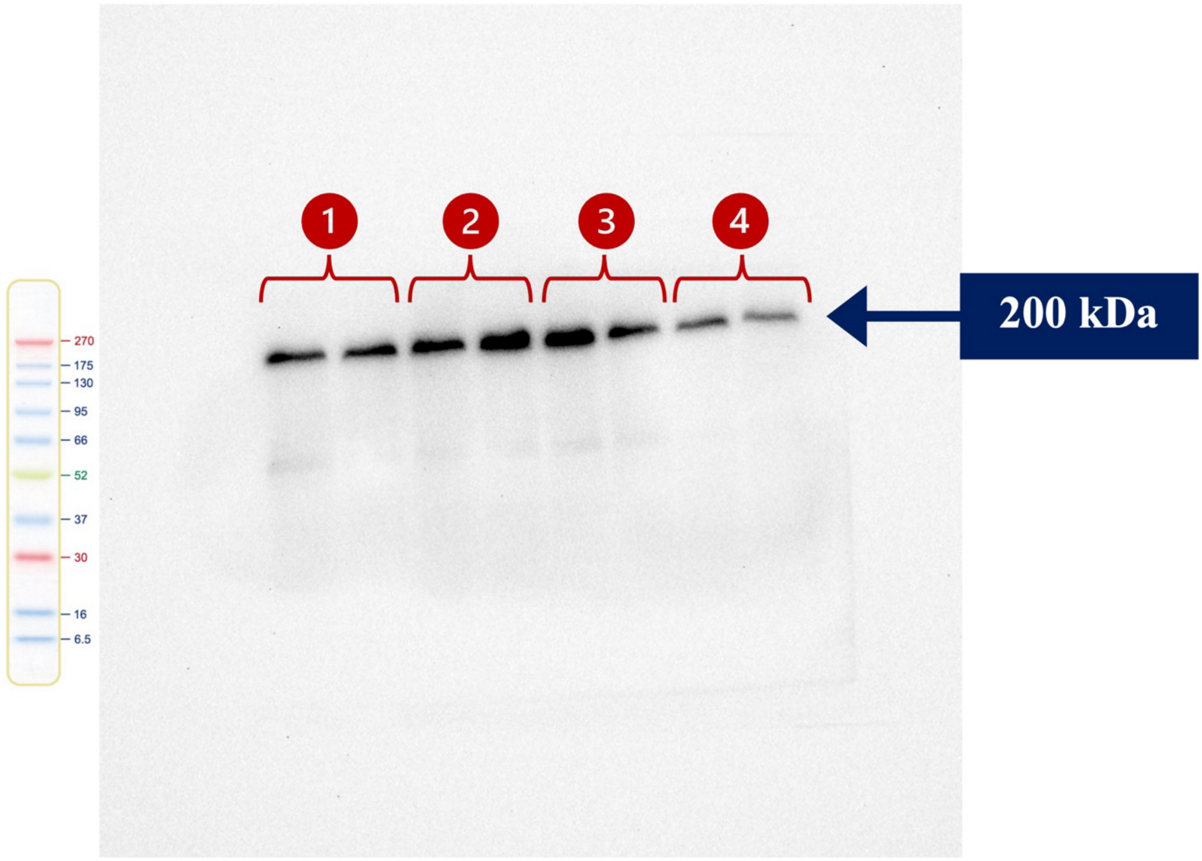

Figure S3: Western blot analysis of DNMT1 expression in SH-SY5Y cells following treatment with curcumin-enriched turmeric extract combined with L-ascorbic acid against hydrogen peroxide-induced neurotoxicity. The experimental groups included: (1) naïve control, (2) hydrogen peroxide + vehicle, (3) hydrogen peroxide + curcumin-enriched turmeric extract combined with L-ascorbic acid (20 µg/mL), and (4) hydrogen peroxide + curcumin-enriched turmeric extract combined with L-ascorbic acid (40 µg/mL). This figure presents the original, unprocessed, uncropped, full-length membrane images.

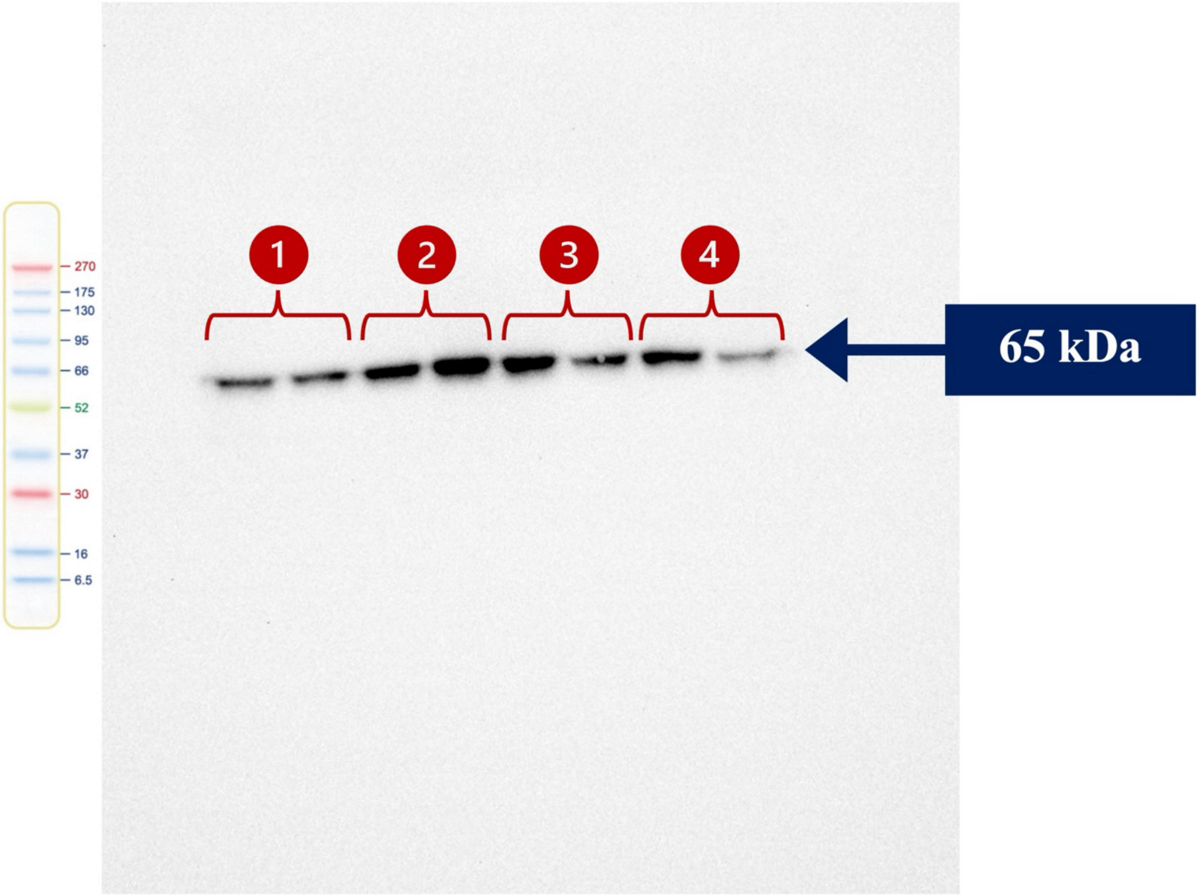

Figure S4: Western blot analysis of NF-κB expression in SH-SY5Y cells following treatment with curcumin-enriched turmeric extract combined with L-ascorbic acid against hydrogen peroxide-induced neurotoxicity. The experimental groups included: (1) naïve control, (2) hydrogen peroxide + vehicle, (3) hydrogen peroxide + curcumin-enriched turmeric extract combined with L-ascorbic acid (20 μg/mL), and (4) hydrogen peroxide + curcumin-enriched turmeric extract combined with L-ascorbic acid (40 μg/mL). This figure presents the original, unprocessed, uncropped, full-length membrane images.

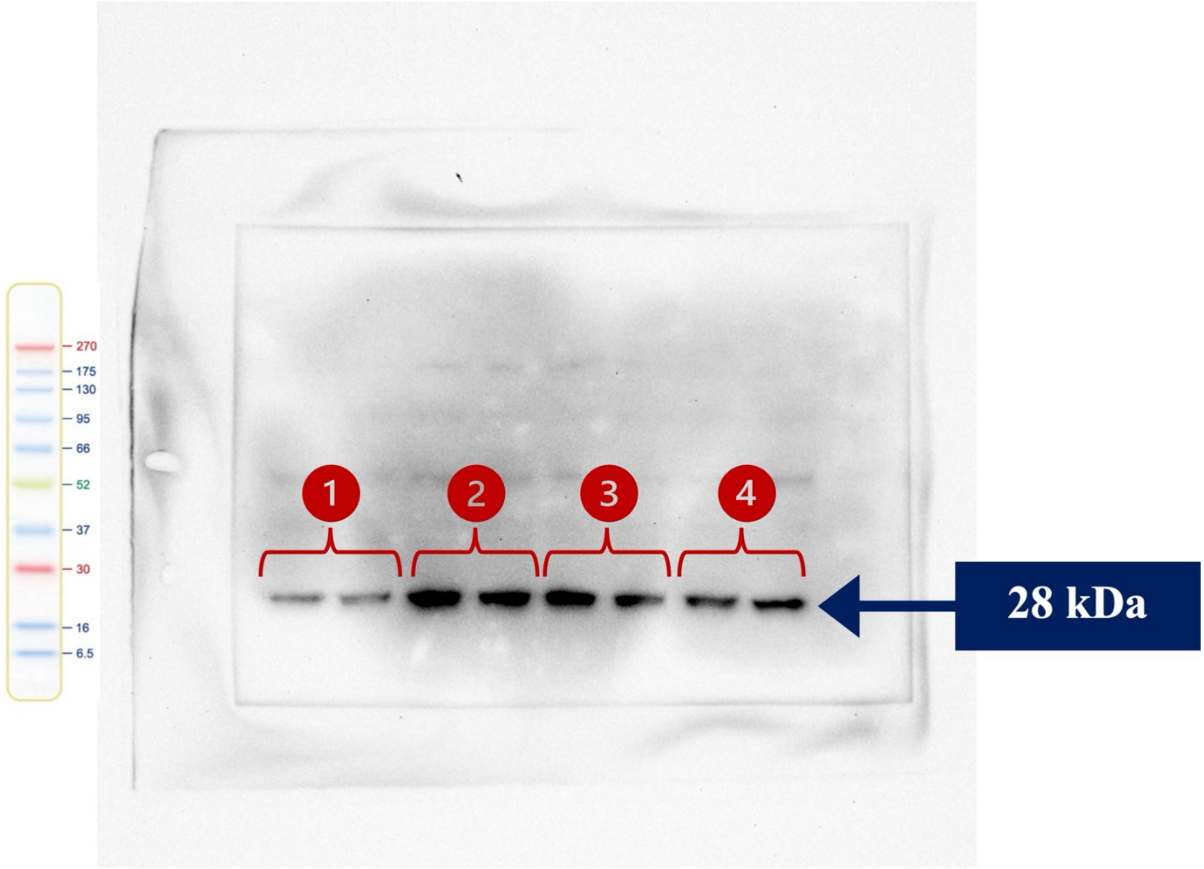

Figure S5: Western blot analysis of IL-6 expression in SH-SY5Y cells following treatment with curcumin-enriched turmeric extract combined with L-ascorbic acid against hydrogen peroxide-induced neurotoxicity. The experimental groups included: (1) naïve control, (2) hydrogen peroxide + vehicle, (3) hydrogen peroxide + curcumin-enriched turmeric extract combined with L-ascorbic acid (20 μg/mL), and (4) hydrogen peroxide + curcumin-enriched turmeric extract combined with L-ascorbic acid (40 μg/mL). This figure presents the original, unprocessed, uncropped, full-length membrane images.

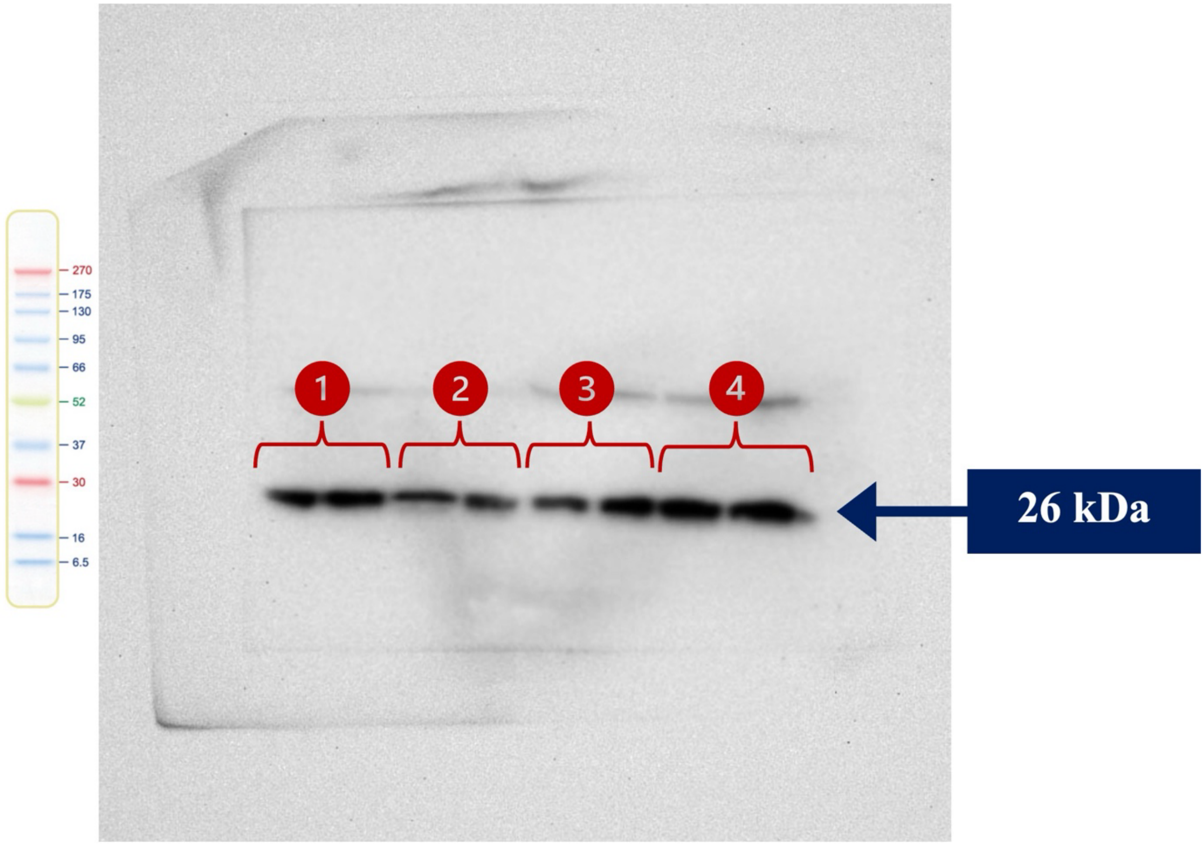

Figure S6: Western blot analysis of Bcl-2 expression in SH-SY5Y cells following treatment with curcumin-enriched turmeric extract combined with L-ascorbic acid against hydrogen peroxide-induced neurotoxicity. The experimental groups included: (1) naïve control, (2) hydrogen peroxide + vehicle, (3) hydrogen peroxide + curcumin-enriched turmeric extract combined with L-ascorbic acid (20 μg/mL), and (4) hydrogen peroxide + curcumin-enriched turmeric extract combined with L-ascorbic acid (40 μg/mL). This figure presents the original, unprocessed, uncropped, full-length membrane images.

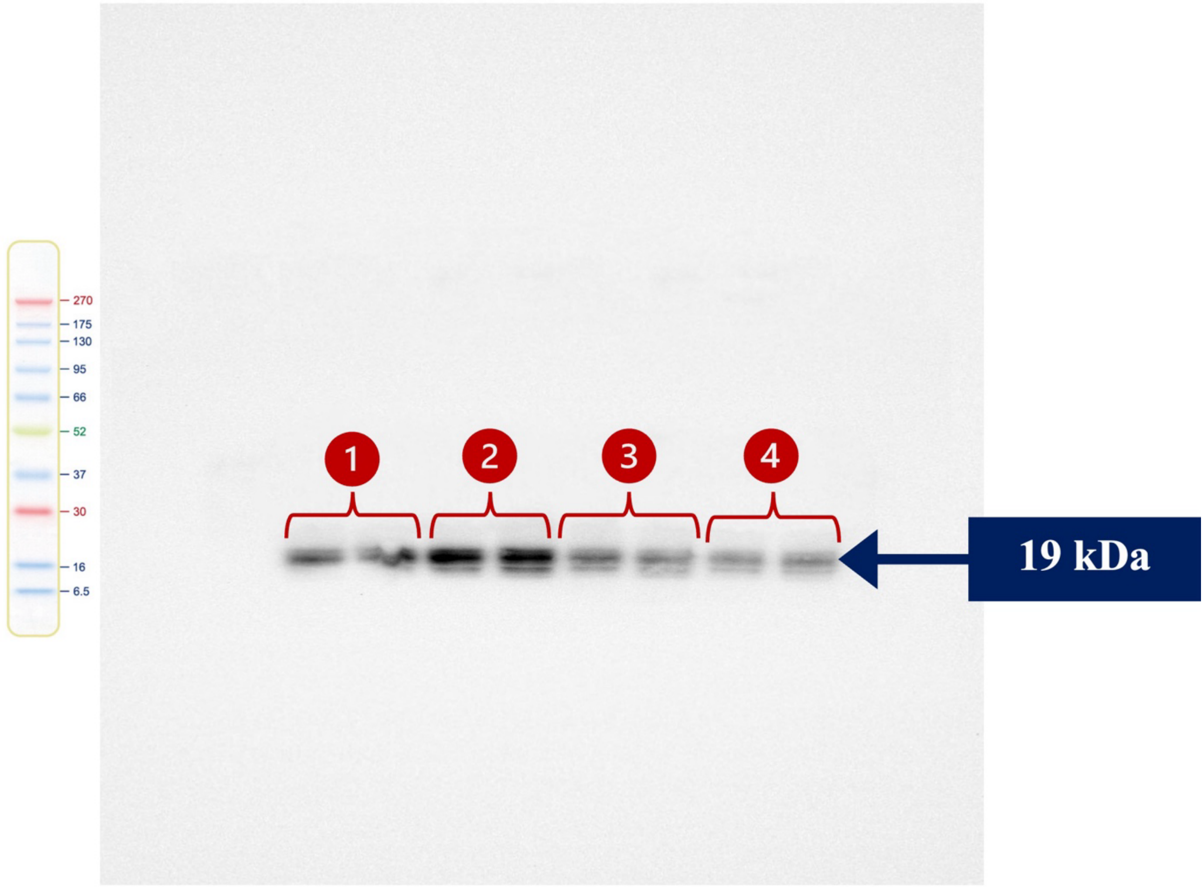

Figure S7: Western blot analysis of caspase-3 expression in SH-SY5Y cells following treatment with curcumin-enriched turmeric extract combined with L-ascorbic acid against hydrogen peroxide-induced neurotoxicity. The experimental groups included: (1) naïve control, (2) hydrogen peroxide + vehicle, (3) hydrogen peroxide + curcumin-enriched turmeric extract combined with L-ascorbic acid (20 µg/mL), and (4) hydrogen peroxide + curcumin-enriched turmeric extract combined with L-ascorbic acid (40 µg/mL). This figure presents the original, unprocessed, uncropped, full-length membrane images.

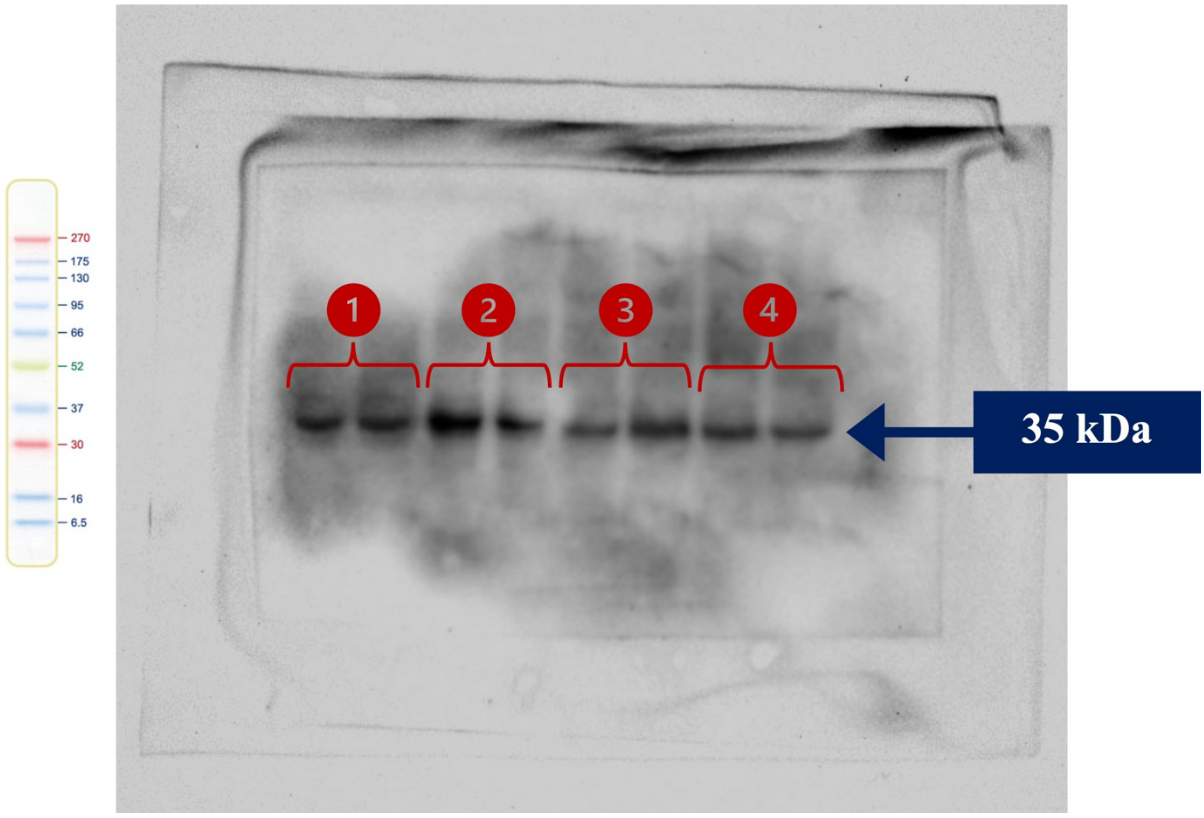

Figure S8: Western blot analysis of caspase-9 expression in SH-SY5Y cells following treatment with curcumin-enriched turmeric extract combined with L-ascorbic acid against hydrogen peroxide-induced neurotoxicity. The experimental groups included: (1) naïve control, (2) hydrogen peroxide + vehicle, (3) hydrogen peroxide + curcumin-enriched turmeric extract combined with L-ascorbic acid (20 μg/mL), and (4) hydrogen peroxide + curcumin-enriched turmeric extract combined with L-ascorbic acid (40 μg/mL). This figure presents the original, unprocessed, uncropped, full-length membrane images.

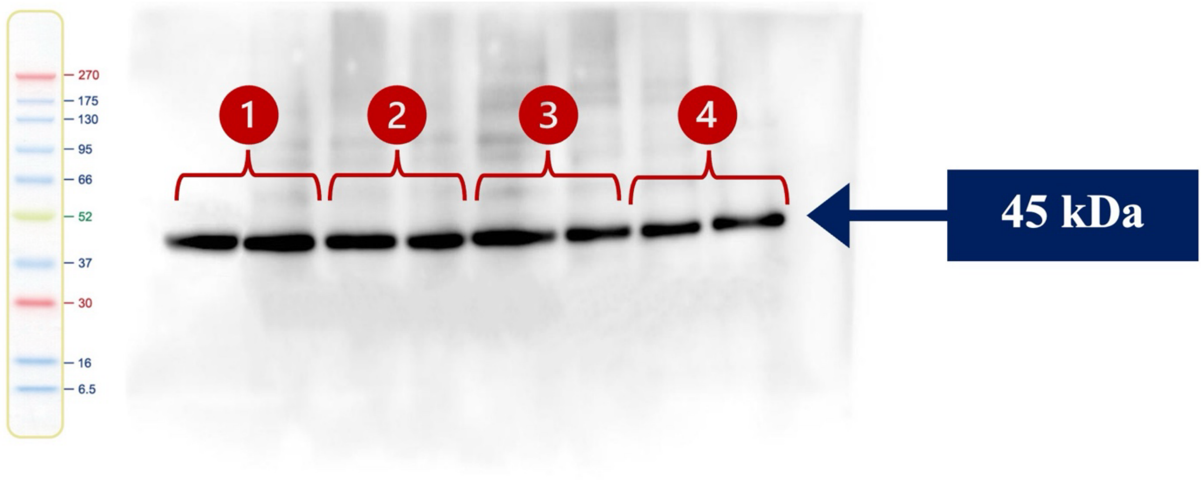

Figure S9: Western blot of β-actin used for the normalization of the relative density of SIRT1 and DNMT1. The experimental groups included: (1) naïve control, (2) hydrogen peroxide + vehicle, (3) hydrogen peroxide + curcumin-enriched turmeric extract combined with L-ascorbic acid (20 μg/mL), and (4) hydrogen peroxide + curcumin-enriched turmeric extract combined with L-ascorbic acid (40 μg/mL). This figure presents the original, unprocessed, uncropped, full-length membrane images.

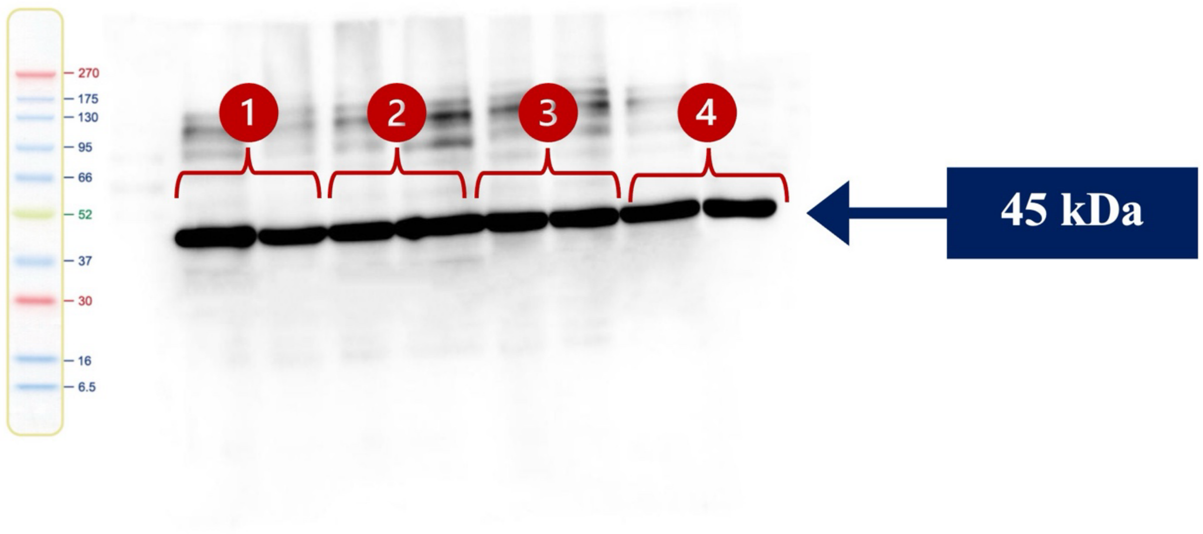

Figure S10: Western blot of β-actin used for the normalization of the relative density of NF-κB and IL-6. The experimental groups included: (1) naïve control, (2) hydrogen peroxide + vehicle, (3) hydrogen peroxide + curcumin-enriched turmeric extract combined with L-ascorbic acid (20 μg/mL), and (4) hydrogen peroxide + curcumin-enriched turmeric extract combined with L-ascorbic acid (40 μg/mL). This figure presents the original, unprocessed, uncropped, full-length membrane images.

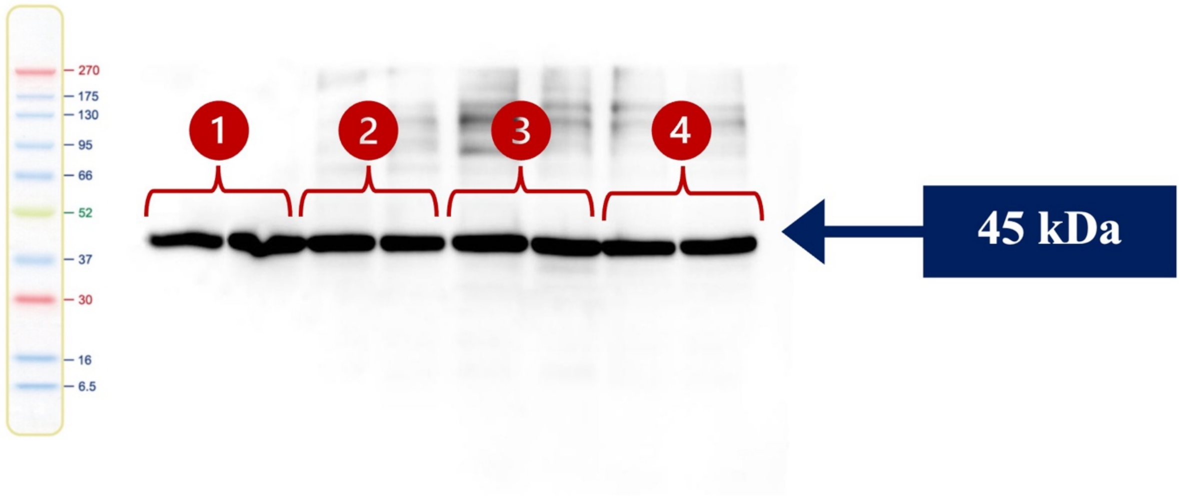

Figure S11: Western blot of β-actin used for the normalization of the relative density of Bcl-2, caspase-3, and caspase-9. The experimental groups included: (1) naïve control, (2) hydrogen peroxide + vehicle, (3) hydrogen peroxide + curcumin-enriched turmeric extract combined with L-ascorbic acid (20 µg/mL), and (4) hydrogen peroxide + curcumin-enriched turmeric extract combined with L-ascorbic acid (40 µg/mL). This figure presents the original, unprocessed, uncropped, full-length membrane images.
